# Universal mechanisms of sound production and control in birds and mammals

**DOI:** 10.1038/ncomms9978

**Published:** 2015-11-27

**Authors:** C.P.H Elemans, J.H. Rasmussen, C.T. Herbst, D.N. Düring, S.A. Zollinger, H. Brumm, K. Srivastava, N. Svane, M. Ding, O.N. Larsen, S.J. Sober, J.G. Švec

**Affiliations:** 1 Department of Biology, University of Southern Denmark, Campusvej 55, 5230 Odense, Denmark; 2 QuanTM program, Emory University, Atlanta, Georgia 30322, USA; 3 Université de Saint-Etienne/Lyon, ENES/CNPS CNRS UMR8195, Saint-Etienne 42023, France; 4 Faculty of Science, Department of Biophysics, Voice Research Lab, Palacky University, Olomouc 77146, Czech Republic; 5 Communication and Social Behaviour Group, Max Planck Institute for Ornithology, Seewiesen 82319, Germany; 6 Department of Biology, Emory University, Atlanta, Georgia 30332, USA; 7 Coulter Department of Biomedical Engineering, Georgia Institute of Technology, Atlanta, Georgia 30332, USA; 8 Department of Orthopaedic Surgery & Traumatology, Odense University Hospital, University of Southern Denmark, Odense 5230, Denmark

## Abstract

As animals vocalize, their vocal organ transforms motor commands into vocalizations for social communication. In birds, the physical mechanisms by which vocalizations are produced and controlled remain unresolved because of the extreme difficulty in obtaining *in vivo* measurements. Here, we introduce an *ex vivo* preparation of the avian vocal organ that allows simultaneous high-speed imaging, muscle stimulation and kinematic and acoustic analyses to reveal the mechanisms of vocal production in birds across a wide range of taxa. Remarkably, we show that all species tested employ the myoelastic-aerodynamic (MEAD) mechanism, the same mechanism used to produce human speech. Furthermore, we show substantial redundancy in the control of key vocal parameters *ex vivo*, suggesting that *in vivo* vocalizations may also not be specified by unique motor commands. We propose that such motor redundancy can aid vocal learning and is common to MEAD sound production across birds and mammals, including humans.

## Abstract (web summary)

In contrast to the larynx of mammals, birds produce sound using a unique vocal organ called the syrinx. Using *ex vivo* preparations, Elemans *et al.* show that, despite large anatomical differences, sound production across a range of avian taxa is via the myoelastic-aerodynamic mechanism, the same mechanism involved in human speech.

## Graphical abstract

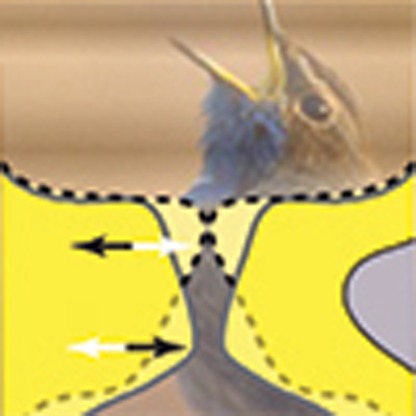

In contrast to laryngeally vocalizing mammals, ∼10,000 species of extant birds vocalize with a uniquely avian vocal organ, the syrinx, located at the tracheobronchial junction and suspended in an air sac of the respiratory system^1^. The syrinx is structurally highly diverse across species^1^, but how morphological diversity reflects functional diversity remains unexplored. In addition, while songbirds are a widely used experimental animal model for neural mechanisms underlying vocal imitation learning^2^,^3^, we lack the empirical evidence to precisely map motor function onto neural circuitry^4^. Addressing these questions requires empirical quantification of syringeal dynamics as a function of control parameters in different species under physiologically realistic, controlled conditions. However, imaging the syrinx *in vivo* remains a challenge^5^,^6^, and we thus still lack this quantification of syringeal dynamics and control parameters.

Earlier endoscopic imaging identified syringeal vibratory tissues in songbirds and non-songbirds^5^,^6^, arguing against purely aerodynamical whistle mechanisms in which sound is produced without periodic movement of the vocal apparatus. Many mathematical models for birdsong^7^,^8^,^9^,^10^,^11^ assume that syringeal sound production is based on a myoelastic-aerodynamic (MEAD) system^12^,^13^,^14^,^15^. However, conclusive empirical evidence for MEAD is lacking^4^,^16^. The MEAD framework explains the physical mechanism underlying laryngeal sound production in mammals^12^,^13^,^14^,^15^,^17^. In brief, self-sustaining laryngeal vocal fold oscillations are maintained through fluid-tissue interactions and (myo)elastic restoring forces generated within the tissues^12^,^13^,^14^,^15^, preventing the need for muscle contractions at the rate of tissue vibration or other periodic input^18^. Expiratory airflow is mechanically converted by vocal folds into pulse-like airflow, which causes air pressure disturbances constituting the acoustic excitation of the system^13^. The mechanical properties and recruitment of different layers of vibrating tissues affect their resonance properties, which in combination with aerodynamic driving forces determine the frequency and mode of oscillation^19^,^20^,^21^,^22^.

According to MEAD theory, the medio-lateral vibration of the inner vocal fold surface (vibrational component 1 (VC1)) that gates airflow^23^ can only be self-sustaining if moving around a stable equilibrium position and if no net energy loss occurs per oscillation cycle^13^,^24^. The latter requires the presence of an aerodynamic force that changes magnitude with direction of vocal fold motion and thus is asymmetric over the oscillation cycle^13^. One possibility is that during self-sustained vocal fold vibration the required asymmetric aerodynamic force is produced by time-varying supraglottal pressure due to inertia of air in the vocal tract^23^. Such a mechanism would result in uniform medio-lateral vibration of the vocal folds^24^. However, models suggest that this mechanism limits the range of fundamental frequencies (F0) produced^14^. Another, more robust possibility is that the asymmetric aerodynamic force during self-sustained vocal fold vibration is produced by out-of-phase motion of the superior and inferior edge of the vibrating tissue (vibrational component 2 (VC2))^13^,^25^. VC1 and VC2 are the respective medio-lateral and caudo-cranial components of a tissue surface wave, or mucosal wave, that travels on the inner vocal fold surface along the expiratory air stream and facilitates aerodynamic energy transfer into tissue^24^. The wave phase changes cause the vocal folds to change shape from convergent during opening to divergent during closing parts of the cycle^24^. Because the intraglottal pressure is higher for the convergent shape than for the divergent shape^26^,^27^, the vocal folds are pushed apart during opening and pulled together during closing^14^,^24^,^25^. VC2 presence thus indicates that intraglottal pressure forms the asymmetric forcing function over opening and closing phases of vibration^25^ essential to self-sustained oscillation. Sound excitation events in mammals occur mainly at glottal closure and/or opening, when airflow abruptly stops or starts^28^,^29^,^30^.

Although isolated aspects of syringeal dynamics have been studied in birds^5^,^6^,^31^,^32^,^33^,^34^,^35^, the asymmetric forcing function essential to maintain self-sustained oscillation has not yet been identified, and the caudo-cranial tissue-wave component VC2—a crucial underlying assumption in modelling studies^7^,^9^,^36^—has not been demonstrated experimentally in the intact syrinx under appropriate physiological conditions^16^. Furthermore, it is unknown how syringeal dynamics relate to sound generating events within a single oscillatory cycle. These essential features of syringeal dynamics required to confirm MEAD have yet to be established^16^.

The translation of vocal motor commands into acoustic output depends on neural activity, musculature, morphology and physical mechanism of sound production^4^,^16^,^37^. These variables define a multi-dimensional parameter space that the brain needs to explore and navigate to control vocal output. In birds, these control variables have been studied *in vivo* by correlating acoustical parameters, for example, F0, with physiological parameters, for example, lung pressure or muscle activity, taking advantage of the highly stereotyped vocal patterns employed by adult birds^38^. This approach can inform us about a particular solution an individual uses to control its vocal output. However, if the control space is redundant (that is, there is more than one possible solution to achieve a specific vocal target, for example, F0), as commonly observed in motor control systems^39^, studying stereotyped *in vivo* behaviour provides limited insight in the behaviour of the entire system as we only observe the final solution the individual uses that may not be unique. Furthermore if vocal control parameters covary it may be difficult to establish causal relationships. To understand how the brain controls vocal behaviour therefore requires systematic quantification of the system's behaviour across its multi-dimensional parameter space. However, we currently lack an experimental paradigm to systematically study physiological control of the vocal system in birds.

Here, we present a novel *ex vivo* paradigm of the syrinx, which allows unprecedented experimental control and high-resolution imaging during sound production. First, to investigate if the MEAD physical mechanism of self-sustained oscillations as observed in mammals is applicable to birds, we test the hypotheses that a caudo-cranial travelling tissue surface wave is present and that this tissue wave is associated with sound production events. Second, to determine whether vocalizations are encoded by unique motor commands we test the hypothesis that the physiological control space of the syrinx *ex vivo* is redundant for key acoustic parameters. We show that birds employ the MEAD mechanism for sound production and that key vocal parameters exhibit redundant control *ex vivo*. We propose that motor redundancy may accelerate vocal learning and is common to MEAD sound production across birds and mammals.

## Results

### Physical mechanism of sound production

**Figure 1:**
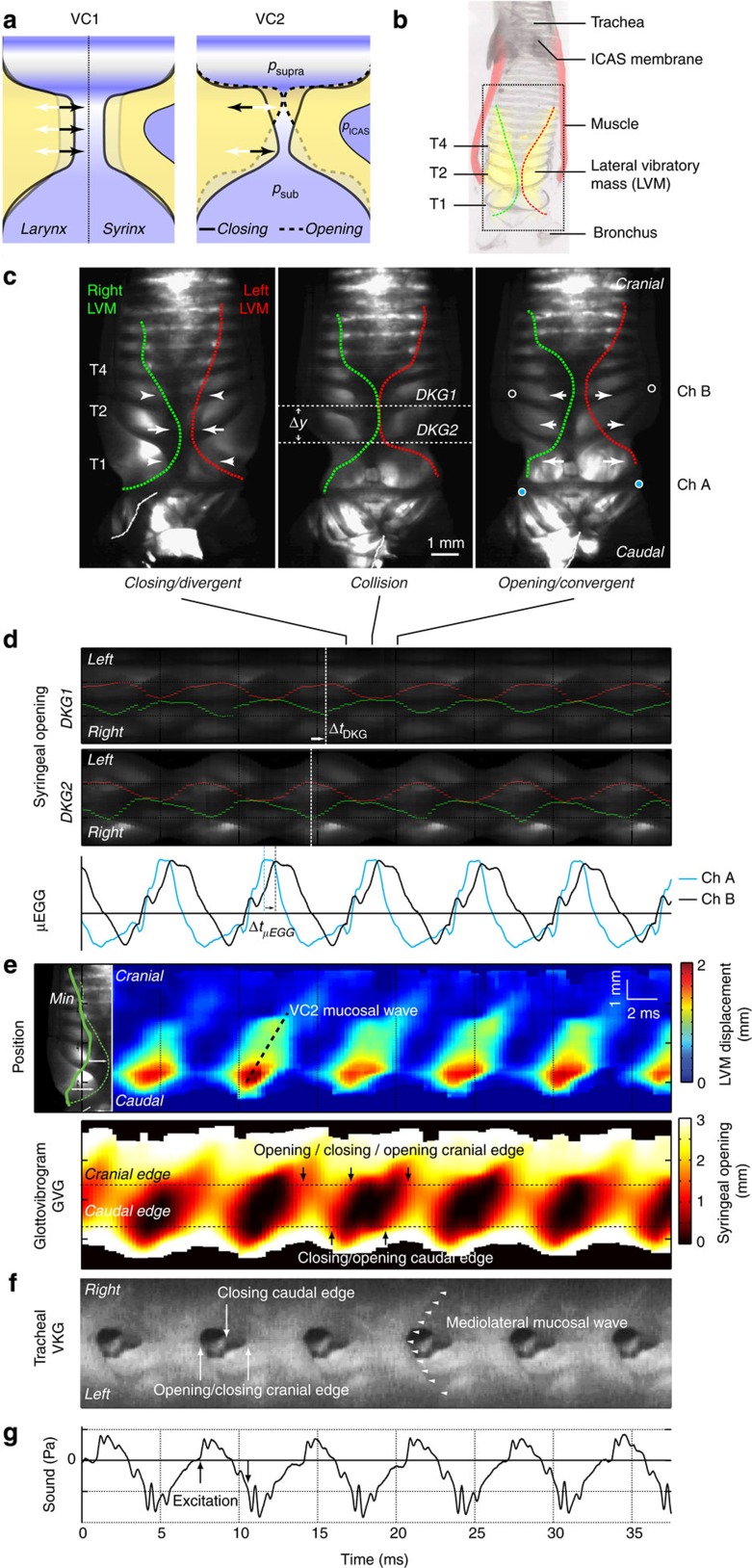
The MEAD theory explains sound production in the domestic pigeon (*Columba livia*) syrinx. (**a**) Schematic illustration of vocal organs in mammals (larynx) and birds (syrinx) showing the two vibrational components essential to MEAD theory: medio-lateral oscillation (VC1), and caudo-cranial phase differences (VC2). The latter are related to a tissue surface wave moving cranially and cause the inner wall of the vibratory tissue to be divergent during closing (solid line, black arrows) and convergent during opening motion (dashed line, white arrows). The syrinx is suspended in the ICAS. Three pressures act on the syrinx: subsyringeal (*p*_sub_), suprasyringeal (*p*_supra_) and ICAS pressure (*p*_ICAS_). (**b**) Annotated micro-CT scan of the pigeon syrinx showing LVM (yellow) and their inner edges (right, green; left, red), muscle (pink) and tracheal rings (T1–4). (**c**) Transilluminated syrinx filmed at 4 kHz with traced outlines of right (green) and left (red) LVM during closing, collision and opening phases of oscillation. The region shown is indicated by the dotted box in **b**. White arrows indicate movement. Circles indicate μEGG electrode pairs channel A (cyan fill) and B (black fill). (**d**) Two DKG (compiled at dotted lines in **c**) and simultaneous μEGG recordings show consistent caudo-cranial time delay of syringeal closure between the caudal line *DKG2* and cranial line *DKG1* (Δ*t*_DKG_) as well as between the caudal and cranial μEGG signals (Δ*t*_μEGG_). (**e**) Spatiotemporal displacement analysis of the left LVM (top) shows a caudo-cranial travelling tissue wave (dashed line), which is also present in the glottovibrogram of syringeal opening (bottom). (**f**) Simultaneous tracheal endoscopic VKG confirms opening and closing event timing of the cranial LVM edge, and reveals tissue waves (arrowheads) travelling mediolaterally over the epithelia. (**g**) Abrupt positive and negative excitation of the acoustic waveform aligns with opening and closing of the LVM cranial edge.

We developed an experimental paradigm that allows imaging of syringeal dynamics under controlled conditions *ex vivo* (Methods section; Supplementary Fig. 1). To test our first hypothesis, if the MEAD physical mechanism of self-sustained oscillations is applicable to birds (Fig. 1a), we took advantage of the diversity in syringeal morphology across species and first studied the domestic pigeon syrinx (Fig. 1b) because of its relatively simple morphology. We found self-sustained syringeal oscillations when both bronchial and air sac pressures were >0.5 kPa (*N*=12). The lateral vibratory masses (LVMs)^7^ were visualized using transillumination of the syrinx (Fig. 1c). Within an oscillation cycle the LVM inner wall changed shape from divergent during closing, rectangular during full collision and to convergent during opening (Fig. 1c, Supplementary Movie 1). A consistent phase shift in LVM position, confirmed by simultaneous micro-electroglottography (μEGG) recordings (Methods section), was present along the caudo-cranial axis (Fig. 1d). Spatiotemporal analysis of the LVM inner wall displacement and syringeal opening identified both components VC1 and VC2 of a tissue travelling wave along the caudo-cranial axis (Fig. 1e). Furthermore, we observed medio-lateral, that is, laterally travelling, tissue waves on the cranial surface of the LVMs (Fig. 1f), which are continuations of the caudo-cranial waves accompanying the LVM opening, and commonly observed on mammalian vocal folds (Supplementary Fig. 2). Sound pressure excitation occurred both during opening and closing events (Fig. 1g). The presence of both VC1 and VC2 components confirms our hypotheses that a caudo-cranial travelling tissue surface wave is present and that this wave is associated with sound producing events in pigeons.

**Figure 2:**
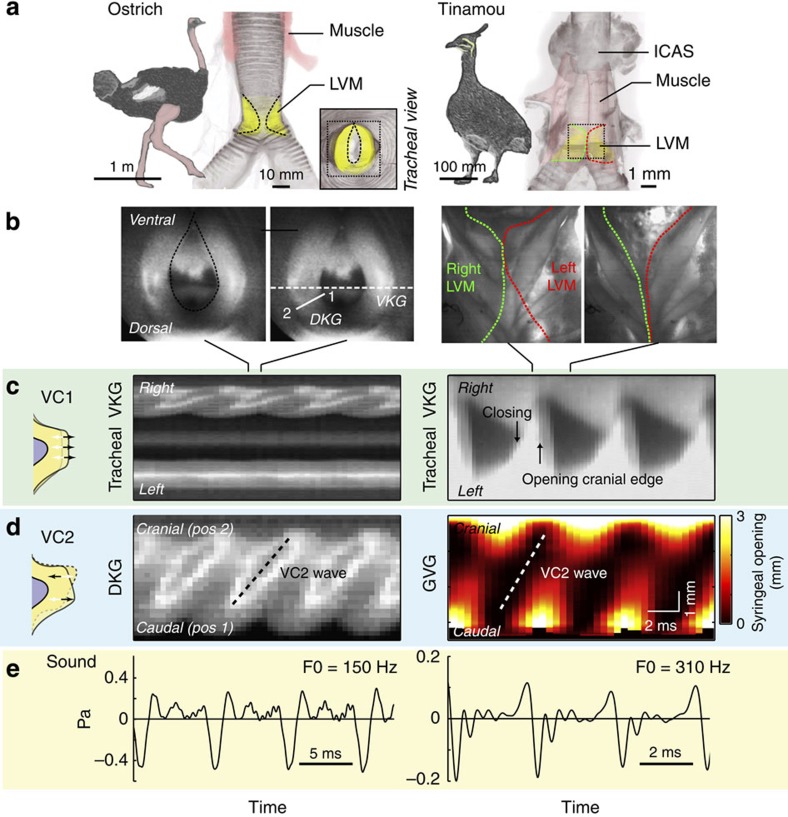
MEAD theory explains syringeal sound production in paleognaths. (**a**) Three-dimensional syrinx geometry of the ostrich (*Struthio camelus*) and elegant-crested tinamou (*Eudromia elegans*) showing bone (grey), LVM (yellow) and muscles (pink). Both views are ventral. Muscle is rendered transparent in tinamou syrinx to show vibratory tissue. (**b**) High-speed imaging stills of endoscopic tracheal (ostrich) and transilluminated frontal views (tinamou) of the syrinx at two different phases during the oscillatory cycle. The region and location of these stills is indicated with a dotted box in **a**. For the ostrich the position of VKG analysis (dotted horizontal line) and DKG analysis (solid white line from point 1–2) presented in panel **c** is indicated. Transillumination provides sufficient detail to identify inner edges of right (green) and left (red) LVM in tinamou. (**c**) Evidence for medio-lateral vibrations (VC1) using tracheal endoscopic VKG. In the ostrich predominantly the right side vibrated. Full opening and closure of the cranial edge can be observed in tinamou. (**d**) Evidence for the caudo-cranial component (VC2) of the tissue wave as demonstrated by DKG for ostrich, and glottovibrogram for tinamou. (**e**) Synchronised acoustic waveforms.

**Figure 3:**
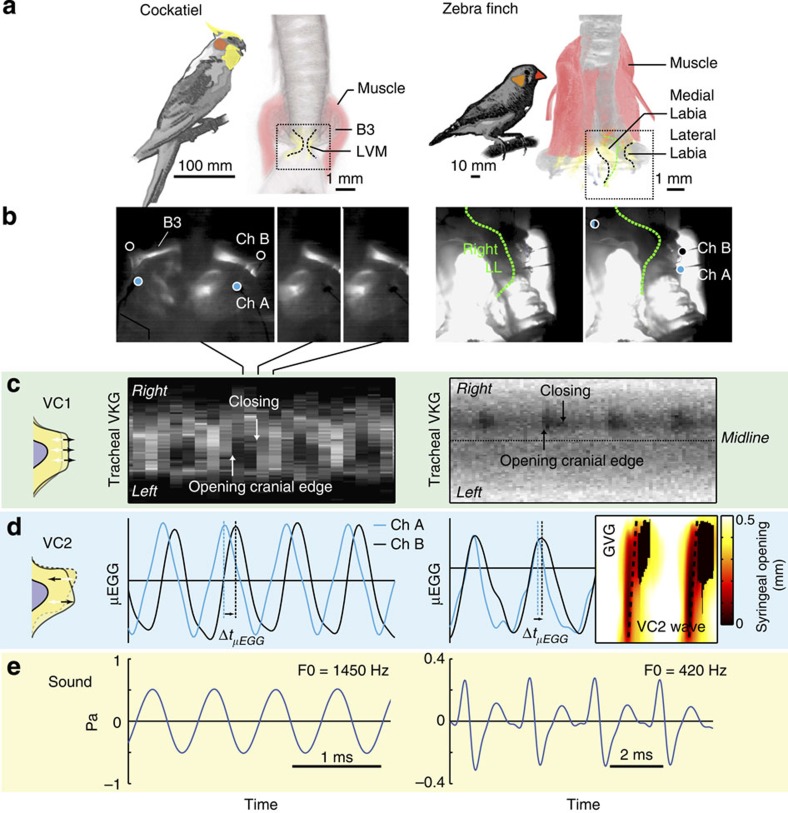
MEAD theory explains syringeal sound production in neognaths. (**a**) Three-dimensional syrinx geometry of the cockatiel (*Nymphicus hollandicus,* ventral view) and zebra finch (*Taeniopygia guttata,* dorsal view) showing bone (grey), LVM (yellow) and muscles (pink). Muscle is rendered transparent in zebra finch right hemisyrinx to show vibratory tissue. (**b**) High-speed imaging stills of frontal transilluminated frontal views of the syrinx at two different phases during the oscillatory cycle at view and location indicated with a dotted box in **a**. While transillumination does not provide detail on motion in the cockatiel syrinx, it provides sufficient detail to identify inner edges of vibratory tissue in female zebra finches (green). Circles indicate location of μEGG electrodes (cyan, channel A; black, channel B). (**c**) Tracheal endoscopic VKG shows opening and closing events and thus evidence for medio-lateral vibrations (VC1). In the zebra finch sound production was induced in the right hemisyrinx. (**d**) Evidence for caudo-cranial component (VC2) of the tissue wave as demonstrated by μEGG phase delays for cockatiel and zebra finch and glottovibrogram analysis for zebra finch. (**e**) Synchronised acoustic waveforms.

To test whether the presence of a tissue surface wave is a shared trait across birds, we studied syringeal oscillatory behaviour in six additional bird species from five orders. We selected species ranging in size (∼15 g–200 kg) and vocal complexity, each with highly divergent syrinx morphologies, containing one or two paired oscillators and controlled by zero to seven pairs of intrinsic syringeal muscles (Figs 2 and 3). We reconstructed three-dimensional geometries including bone, soft tissue and muscle based on micro-computed tomography (CT) scans (Figs 2a and 3a). Our study species included the largest extant bird (ostrich), a phylogenetically basal paleognathid bird (elegant-crested tinamou) and several neognathids: a parrot (cockatiel), dove (Barbary dove) and two songbirds (zebra finch and Bengalese finch). Medio-lateral vibration components (VC1) were observed in each species using tracheal endoscopy (Figs 2c and 3c). These VC1 vibrations resulted in complete syringeal closure within an oscillatory cycle in each species except for the ostrich, where full closure was never observed, more closely resembling human breathy phonation^40^.

**Figure 4:**
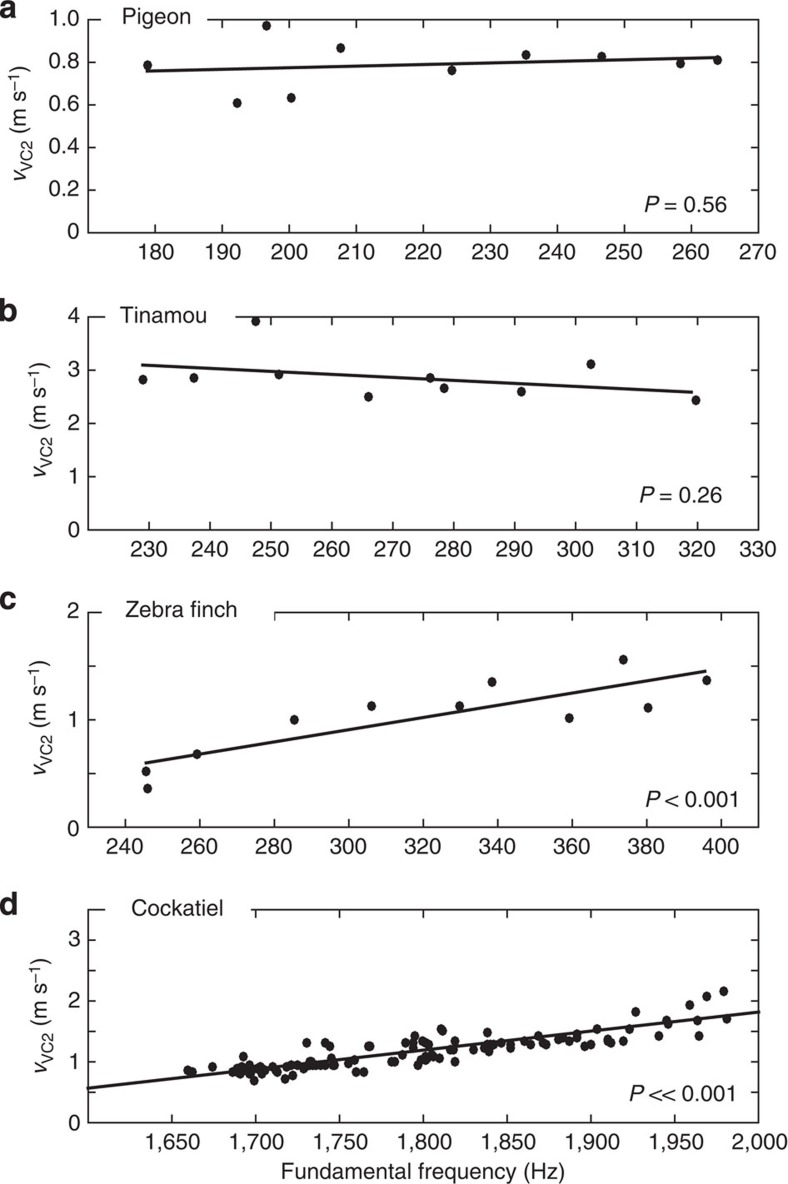
The caudo-cranial tissue wave is present across a range of fundamental frequencies. Magnitude of the caudo-cranial component (*v*_VC2_) of the travelling tissue-wave's velocity in (**a**) pigeon and (**b**) tinamou, (**c**) zebra finch and (**d**) cockatiel. The positive velocity values indicate that the wave travelled from caudal to cranial. Wave speed remained constant with F0 in pigeon and tinamou (Linear regression, *P*=0.56 (*n*=10) and *P*=0.26 (*n*=10), respectively), but increased significantly with F0 in zebra finch and cockatiel (linear regression, *P*<0.001 (*n*=11) and *P*<<0.001 (*n*=118), respectively).

In each species, regardless of syringeal morphology, we confirmed the presence of the caudo-cranial component (VC2) of a travelling tissue wave using spatiotemporal analysis of syringeal inner wall displacement, syringeal opening and/or μEGG (Figs 2d and 3d, Supplementary Movies 1–6). To further test if the tissue wave was present over a range of fundamental frequencies, we subjected the syrinx to bronchial pressure ramps and measured the direction and speed of the tissue wave when present (Methods section). During self-sustained oscillations over a range of F0 values, the tissue wave was always present (Fig. 4) and running from caudal to cranial (indicated by all velocities being positive in Fig. 4). The wave speed did either not vary (pigeon and tinamou; linear regression, *P*=0.56 (*n*=10) and *P*=0.26 (*n*=10), respectively) or increased significantly with F0 (zebra finch and cockatiel; linear regression, *P*<0.001 (*n*=11) and *P*<<0.001 (*n*=118) respectively) within a range of 0.5–4.0 m s^−1^.

**Figure 5:**
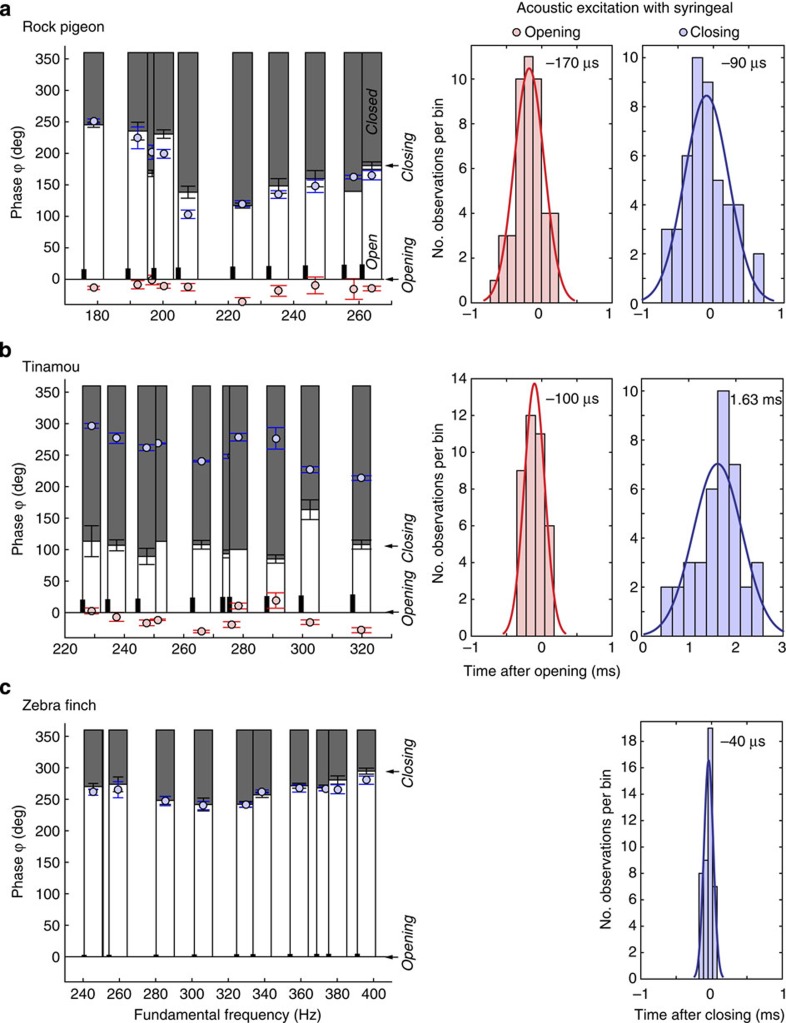
Sound excitation is associated with syringeal opening and closing events. The relative duration of the syrinx in open (white bar starting at phase *ϕ*=0°) and closed (grey bar) state within a single oscillatory cycle (full oscillation equals 360°) over a range of fundamental frequencies in (**a**) pigeon, (**b**) tinamou and (**c**) zebra finch (left) based on transillumination data (Methods section). Sound excitation events are associated with opening (red circles: mean±s.d.) and/or closing (blue circles: mean±s.d.) of the syrinx. The thick vertical black line at the base of each column indicates the duration of a single movie frame relative to the cycle duration and thus the timing accuracy for determining a syringeal closing or opening event. Note that in zebra finch the lines are short due to high frame-rates used (25 kHz). The histograms (right) show that timing of sound excitation is closely associated with syringeal opening (red) and closing (blue) events.

Furthermore, to investigate the relationship between tissue motion dynamics and sound generation events within a single oscillatory cycle, we quantified the delay between sound excitation events and first opening and closing events of the syringeal passage over a range of F0 values (Fig. 5). In the pigeon, both opening and closing events were precisely accompanied by an acoustic excitation at very short delays of 170 and 90 μs, respectively (Fig. 5a), which are both below the 250 μs temporal accuracy of the opening and closing event timing (that is, one frame duration of high-speed video). The tinamou showed a strong acoustic excitation on syringeal opening, with a delay of 100 μs, also below the 250 μs temporal accuracy of the opening and closing event timing (Fig. 5b). A second, often weaker, excitation occurred 1.63 ms after closing. The zebra finch showed a very precisely timed strong acoustic excitation on syringeal closing at a delay of 40 μs (at a temporal accuracy of 33 μs; Fig. 5c).

In conclusion, these data confirm our first hypothesis that a caudo-cranial travelling tissue surface wave is present across a range of syringeal morphologies and sizes. Furthermore, our data confirm a close association between opening/closing event timing and sound generation events within single oscillatory cycles across a range of species. Collectively, our data thus provide the essential lacking demonstrations of syringeal dynamics and sound generation events required to conclusively show that MEAD theory is applicable to sound production in birds.

### Vocal control redundancy *ex vivo*

**Figure 6:**
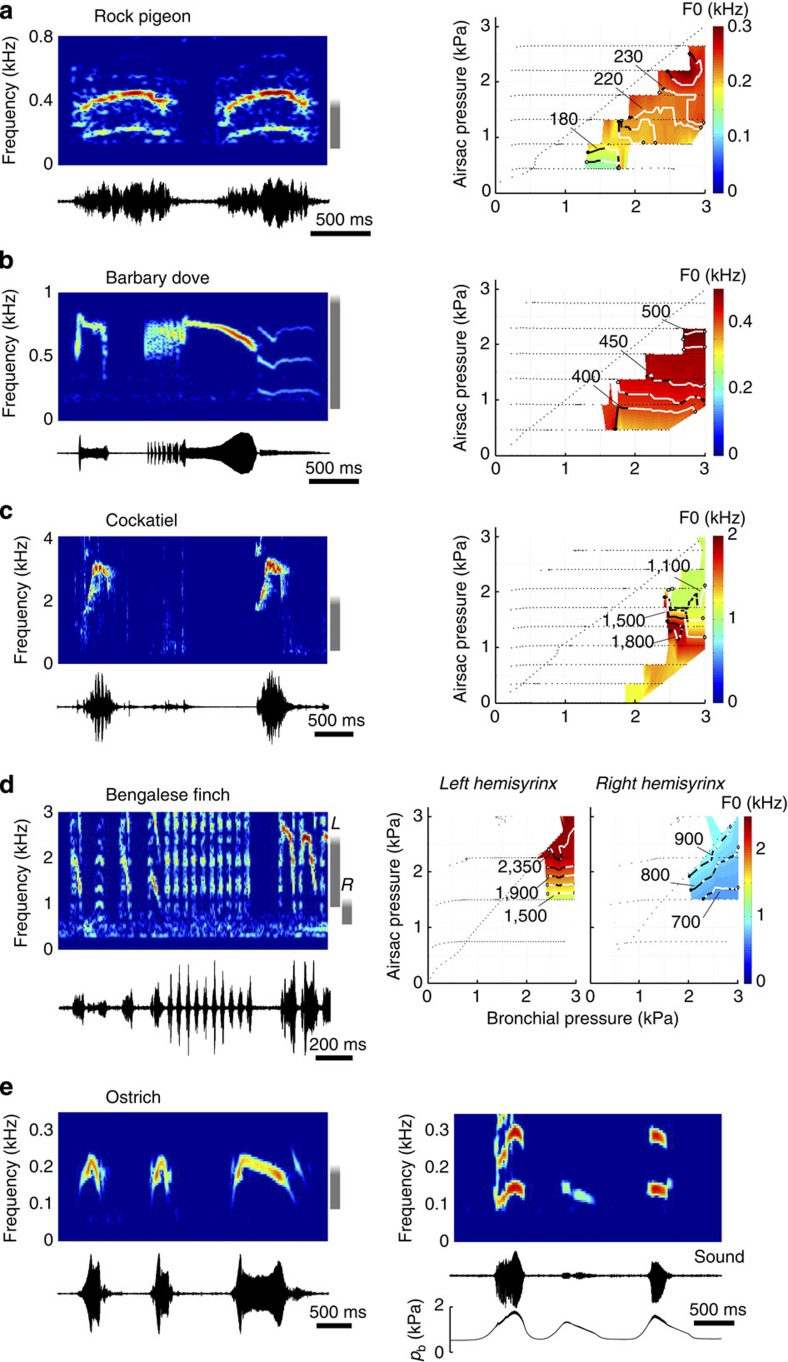
Pressure control of F0 in sound production is redundant across avian taxa. Spectrograms and oscillograms of spontaneous vocalisation (left) and representative pressure control spaces *ex vivo* (right) of (**a**) pigeon (*Columba livia*), (**b**) Barbary dove (*Streptopelia risoria*), (**c**) cockatiel and (**d**) Bengalese finch (*Lonchura striata domestica*). The F0 ranges of sounds produced *ex vivo* (grey vertical bar right of spectrogram) correspond well to the lower range of spontaneous vocalizations. Pressure control may allow higher F0 values, as indicated by the grey bar fading out. Iso-F0 contours (values in Hz) are overlaid on the pressure control spaces and shown in white when redundant for all three acoustics parameters (F0, SPL and WE), or black when not. In the Bengalese finch, the left hemisyrinx produces higher F0 than the right hemisyrinx, corroborating earlier *in vivo* studies^70^. (**e**) Spectrogram and oscillogram (left) of ostrich mating call, and *ex vivo* sound production including bronchial pressure (*p*_b_) patterns underlying phonation (right). A complete pressure control space was not obtained for this species.

**Figure 7:**
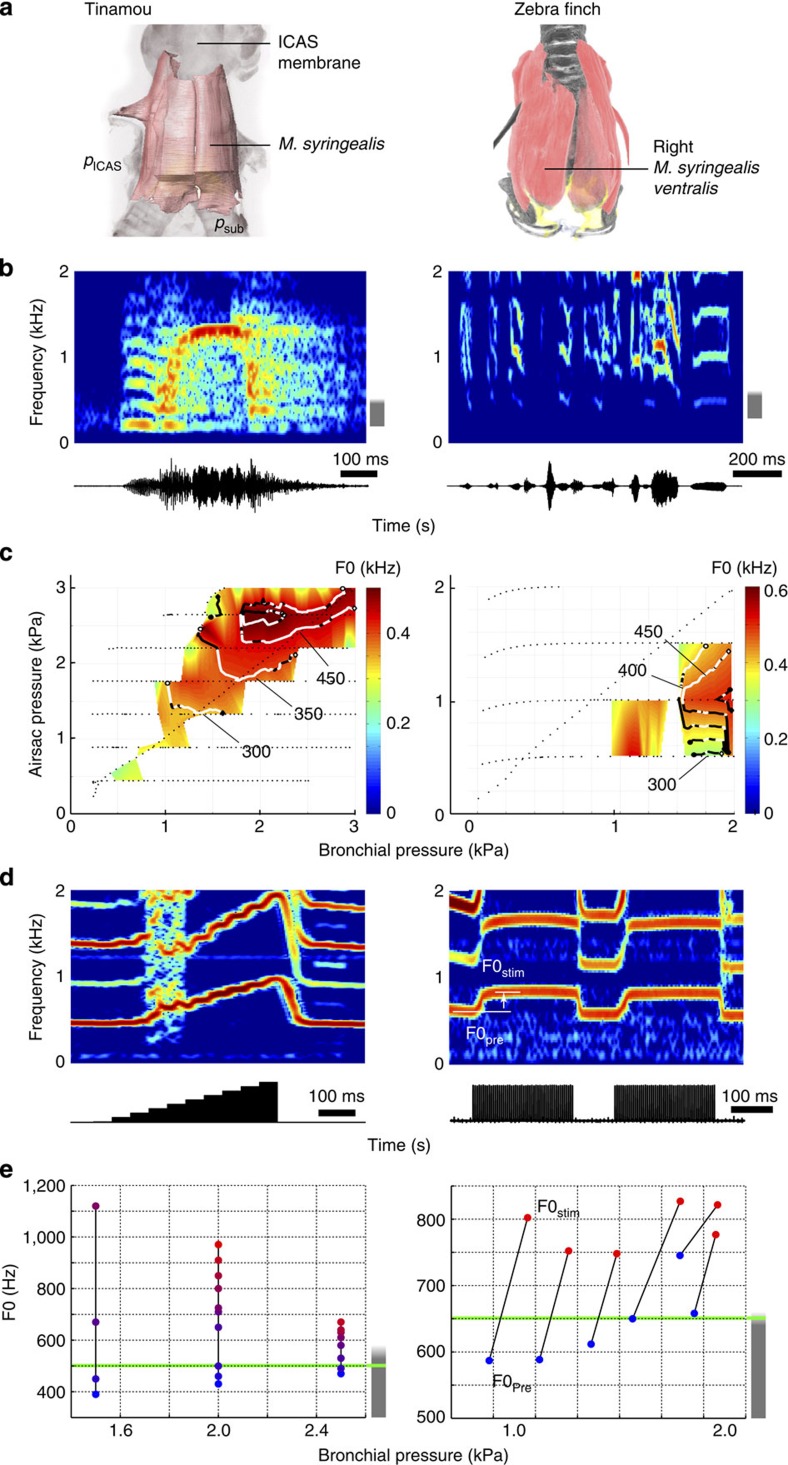
Motor control of F0 in sound production is redundant. (**a**) Three-dimensional geometry of the tinamou and zebra finch syrinx with pressure control parameters; bronchial or subsyringeal (*p*_sub_), and ICAS pressure (*p*_ICAS_). Stimulated muscles are indicated. (**b**) Sound spectrogram (top) and oscillogram (bottom) of a tinamou honksqueal call and zebra finch song motif. (**c**) Representative pressure control spaces *ex vivo* of one individual. Iso-F0 contour as in previous figure (values in Hz). (**d**) Sound spectrogram (top) and muscle stimulus (bottom) during syringeal muscle stimulation. Black dots indicate measurements points. (**e**) Fundamental frequency (F0) is affected by muscle stimulation (blue dots, no stimulation; red dots, maximal stimulation) and pressure. The grey bar indicates the individuals' frequency range achieved by varying only pressure. Pressure control may allow higher F0 values, as indicated by the grey bar fading out. Multiple different combinations result in the same frequency, for example, 500 and 650 Hz in tinamou and zebra finch, respectively, (green horizontal line).

To test our second hypothesis that the physiological control space of the vocal organ is redundant for key acoustic parameters, we systematically investigated the relationship between syringeal control parameters and acoustic output *ex vivo*. In mammals, the brain can control bronchial pressure and laryngeal muscle activity to modulate aerodynamic forces and vocal fold tissue properties (for example, geometry and elasticity) and attain a target fundamental frequency (F0)^12^,^13^,^14^,^15^. In birds, in addition to bronchial pressure, pressure in the interclavicular air sac (ICAS) enclosing the syrinx also affects F0, as suggested by models^7^,^9^,^10^ and experimental manipulations^10^. We first quantified syringeal oscillatory behaviour in different species as a function of bronchial and air sac pressure (Figs 6 and 7a–c). For each species the F0 ranges produced *ex vivo* corresponded well to the lower-end distribution of spontaneous vocalizations (Figs 6 and 7b,c). We further found that in each investigated species, multiple different combinations of bronchial and ICAS pressures could achieve the same target frequency (iso-F0 contours in Figs 6 and 7c), indicating that F0 control is redundant within the pressure control space. To investigate how much two other important vocal parameters, that is, sound pressure level (SPL) and sound quality, changed with F0, we quantified the SPL and Wiener entropy (WE) along evenly spaced iso-F0 contours. A point on the iso-F0 contour was considered to be redundant in all three acoustic parameters if neither SPL nor WE changed along the iso-F0 contour (Methods section). The percentage of the explored control space that demonstrated redundancy for the three vocal parameters measured 79.8±21.4% (mean±s.d.) for pigeon (*N*=7), 83.0±7.7% for Barbary dove (*N*=3), 67.8±4.7% for tinamou (*N*=3), 71.4±18.9% for cockatiel (*N*=2), 44.3% for zebra finch right hemisyrinx, 62.4±13.3% for Bengalese finch left hemisyrinx (*N*=6) and 71.6±18.6% for Bengalese finch left hemisyrinx (*N*=6). These results demonstrate that F0 control by pressure *ex vivo* is redundant in subspaces within the pressure control space, and that this redundancy is conserved across species exhibiting a wide range of syringeal morphologies. Therefore, in the *ex vivo* preparation redundant acoustic output can be achieved by modulating air pressure, without any active control of syringeal muscles.

We furthermore examined whether muscular control introduced an additional source of redundancy and quantified the effect of syringeal muscle recruitment on F0 using local micro-stimulation (Fig. 7d). Muscle stimulation caused an F0 increase of 300–750 Hz and 75–200 Hz for tinamou and zebra finch, respectively (Fig. 7e). The F0 range achieved by pressure and muscle recruitment overlapped within each species (tinamou (*N*=1) and zebra finch (*N*=5)) and multiple different combinations of muscle recruitment and bronchial pressure resulted in the same F0. These results thus demonstrate that muscular control of F0 is redundant in the *ex vivo* preparation.

Taken together, the above data confirm our second hypothesis that the physiological control space of the vocal organ *ex vivo* is redundant for key acoustic parameters across a range of syringeal morphologies and sizes.

## Discussion

Our data establish that birds use MEAD as the primary physical mechanism for sound production with strong similarities to mammalian MEAD systems: First, we demonstrate the presence of a tissue wave that travels from the caudal to cranial end of the syringeal vibratory tissue (Figs 1, 2, 3, 4). This tissue wave thus causes the syringeal vibratory tissue shape to be convergent when opening, and divergent when closing during expiratory sound production, and can be considered analogous to the caudo-cranial mucosal wave observed in mammalian vocal folds. Our data therefore strongly suggest that in birds, just as in mammals, the dominant asymmetric forcing function over the opening and closing phases of vibration essential to maintain self-sustained oscillation is not formed by the mass inertance of the air column in the vocal tract^23^,^24^, but by the tissue-wave-induced intraglottal pressure changes. This mechanism reduces the dependency of the self-sustained syringeal oscillations on acoustic resonances of the vocal tract and allows for an expanded F0 range of vocalization^14^, which in addition to labial morphology^41^ could aid birds in extending their F0 range. Second, the magnitude range of the tissue-wave speed measured (0.5–3.0 m s^−1^) is in excellent agreement with values reported for the mammalian larynx^30^,^41^,^42^,^43^,^44^,^45^,^46^ (human^41^,^43^,^44^,^45^, 0.5–2.0 m s^−1^; dog^42^,^46^,^47^, 0.5–2 m s^−1^; calf^45^, 0.4 m s^−1^; and an African elephant^30^, 1.2 m s^−1^) suggesting that no scaling effects occur. Third, our data show that in some species (zebra finch, cockatiel) the tissue-wave speed increased with F0, similar to what has been observed in humans^47^, whereas in other species (pigeon, tinamou) the tissue-wave remained constant with respect to F0 (Fig. 4). The different relationships are possibly related to biomechanical properties of the tissue layers involved in the vibrations or varying aerodynamic forces acting on the vibrating structures. Future work will be required to establish the respective causality. Last, our findings demonstrate that acoustic excitation occurs at opening and/or closing of the syringeal passage (Fig. 5), which strongly suggests a causal relation between tissue vibration and generation of acoustic energy by modulating the glottal airflow as found in the mammalian larynx^28^,^29^,^30^. While in humans the maximum acoustic excitation has been shown to occur mainly at the instant of glottal closure^28^,^29^, a recent study reported that in an excised elephant larynx the maximum acoustic excitation occurred at the instant of glottal opening^30^. The mammalian larynx thus exhibits more diversity than previously thought in what movements cause the predominant acoustic excitation. Our work demonstrates that this diversity is also present in birds and that more comparative studies are needed to explain the causal link between airflow, tissue vibration and acoustic excitation.

In conclusion, we find that despite the large diversity present in syringeal morphology of the bird species included in this study, all use MEAD as the primary physical mechanism of sound production, supporting MEAD-based approaches to modelling avian sound production^7^,^8^,^9^,^10^,^11^. Moreover, our findings suggest that despite their different evolutionary origins^1^, laryngeally vocalizing mammals and syringeally vocalizing birds have converged on the same physical mechanism for vocalization.

Furthermore, our data show that key acoustic parameters, such as particular F0 values, can be achieved by multiple distinct combinations of respiratory pressure and syringeal muscle recruitment *ex vivo* (Figs 6 and 7). This finding thus supports the hypothesis that subspaces within the entire physiological control space of the vocal organ *ex vivo* are redundant for acoustic parameters.

Our work represents the first systematic exploration of syringeal behaviour in a controlled environment using pressure differentials up to 2 kPa. Although it remains unresolved to what degree birds can independently control bronchial pressure and ICAS air sac pressure *in vivo*, ventilation flow across the lungs requires the existence of pressure differences between different parts of the respiratory system^48^,^49^,^50^. Especially in dynamic situations such as vocal behaviour, the passive geometry of the air sac system can lead to substantial pressure differences between air sacs^51^,^52^ and indeed transient pressure differences up to 1.0 kPa occur during vocalizations in Barbary doves^7^,^53^. These pressure fluctuations can result in local transmural pressure differentials over the syringeal walls and thus in transmural forces on the vibrating tissues^7^,^37^. The individual bird can either avoid or exploit such regimes during vocalization. However, regardless of pressure differentials, only bronchial pressure in combination with different levels of recruitment in a single vocal muscle leads to redundancy in F0 (Fig. 7e).

How acoustic redundancy of the vocal organ *ex vivo* reflects the functional motor redundancy *in vivo* remains to be explored. The speed at which consecutive motor commands can be executed *in vivo* (either on the same side of the syrinx or when vocal production switches rapidly from one side to the other within a complex syllable) may cause a significant reduction of the available functional redundancy, especially because at least some songbirds possess superfast syringeal muscles that produce peak force in <5 ms (refs 54, 55). Here, we did not investigate the rapid time-varying pressure and muscle recruitment patterns that occur *in vivo*, and indeed for most species more detailed experimental *in vivo* data are required to meaningfully explore the high-dimensional control space *ex vivo*. On the other hand, we show that redundancy emerges when only varying the activity level of a single vocal muscle. Redundancy can thus be expected to increase when the brain can produce a given acoustic output by choosing from a large set of redundant motor commands when coordinating the ∼16 syringeal muscles^56^ with several thousand motor units^57^.

A recent study used zebra finch song acoustics to infer low-dimensional motor commands^11^, which were then used to generate synthetic songs that closely resembled the acoustic output of natural vocal behaviour, and evoked auditory responses that closely resembled the pattern of premotor activity. These results led the authors to suggest that premotor area HVC encodes a low-dimensional forward model of gesture dynamics^11^. In contrast, our data indicate that particular acoustic outputs can be specified by multiple motor states, suggesting that motor commands cannot be uniquely inferred from acoustics. It will therefore be important for future studies to investigate whether the method used in ref. 11 to infer motor commands from acoustic data yields the actual combination of control parameters employed by the bird to generate the sound, rather than a different set of control parameters that redundantly produces the same acoustic output. Similarly, future *ex vivo* studies employing time-varying stimulus patterns across multiple muscles will allow us to explore the limits of vocal redundancy during sophisticated motor trajectories similar to those in behaving animals. Despite the apparent conflict between our findings and elements of ref. 11, it is important to note that the instantiation of a low-dimensional forward model within HVC—which is multiple synapses upstream from the vocal and respiratory muscles—is not incompatible with a redundant vocal organ. Indeed, one strength of low-dimensional encoding in HVC is that it would allow HVC to represent control parameters (for example, vocal fold tension) without regard to the details of the redundant combinations of muscle contraction states required to achieve that tension.

In many fine motor control systems such as arm reaching, the brain must negotiate the so-called motor redundancy problem^39^, in which a particular behavioural target can be achieved by vast numbers of motor commands. However, one potential advantage to a redundant control space is that it allows the brain to find subspaces of possible motor commands (for example, along the iso-F0 contours in Figs 6 and 7c in our *ex vivo* paradigm) rather than searching for unique motor commands to achieve the target. Crucially, redundant control spaces allow variability in task-irrelevant directions^58^,^59^. In vocal production, the brain may select from within this motor command space to meet other demands, such as acoustic targets in future and/or past syllables. Consequently, we speculate that vocal motor redundancy may simplify trial-and-error learning during song acquisition by allowing the brain to rapidly discover one of many motor solutions^60^ before learning how to further improve performance by exploiting motor redundancy^39^. Vocal redundancy may therefore have aided, or even been necessary for, the evolutionary development of vocal learning. Future studies might evaluate this speculation by quantifying whether learned vocalizations exploit vocal redundancy. Furthermore, because many species do not learn their vocalizations and many vocal learners produce innate vocalizations in addition to learned ones, future work might also determine whether innate vocal motor programs similarly exploit redundancy to improve performance.

Our *ex vivo* results suggest that the redundant control of key vocal parameters represents a significant aspect of avian vocal control, and one that merits further investigation. Because redundancy in F0 control by pressure and muscle recruitment is also observed in various simplified computational models of the human larynx^13^, and songbird syrinx^9^ as well as in *ex vivo* dog larynx preparations^61^,^62^, we propose that vocal control redundancy is a typical feature of MEAD sound production systems and hence a common feature of vocal production and control in mammals and birds.

## Methods

### Subjects

To study sound production mechanisms and the effect of muscle stimulation on sound production, we used 12 adult domestic pigeons (*Columba livia*; order Columbiformes; nine males, three females), four domestic Barbary doves (*Streptopelia risoria*; order Columbiformes; three males, one female), three elegant-crested tinamous (*Eudromia elegans*; order Tinamiformes; two males, one female), two ostriches (*Struthio camelus*; order Struthioniformes; sex unknown), three cockatiels (*Nymphicus hollandicus;* order Psittaciformes; two males, one female), six Bengalese finches (*Lonchura striata domestica*; order Passeriformes; six males) and 19 zebra finches (*Taenopygia guttata*, order Passeriformes; five adult males; two juvenile males (∼40 dph); 12 females). Ostrich syrinxes were obtained from a local breeding farm. They were transferred to ice immediately after extraction, flash frozen in liquid nitrogen and stored at −80 °C. Bengalese finches were kept in indoor aviaries on a 14-h light:10-h dark light cycle with food and water *ad libitum* (Emory University, Atlanta, GA, USA). Zebra finches were kept in indoor aviaries on a 12-h light:dark photoperiod with food and water *ad libitum* (University of Southern Denmark (SDU), Odense, Denmark). All other animals were kept in 3 × 6 × 2 m outdoor aviary with food and water *ad libitum* (SDU, Odense, Denmark). Pigeons, doves, cockatiels and tinamous were obtained from local breeders. Bengalese finch experiments were carried out at Emory University, and all other experiments at SDU, Denmark. All experiments were conducted in accordance with the Institutional Animal Care and Use Committee of Emory University and of SDU.

### Surgical and mounting procedures

Animals were euthanized with isoflurane and cooled on ice or icepacks. The syrinx and associated blood vessels were dissected out using a stereoscope (M165-FC, Leica Microsystems) through a ventral incision to the sternum while regularly flushing with oxygenated Ringers solution (5 °C, recipe cf. refs 54, 55, 56), and transferred to a Sylgard-covered petri dish on ice containing oxygenated Ringer solution. The syrinx was cleaned of fat and connected to species–specific tubing assemblies of non-reactive polyethylene tubing (Instech Salomon, PA, USA) in the experimental chamber with 10/0 nylon suture (S&T, Neuhausen, Switzerland). Special care was taken to mount the syrinx of each species in its natural position by leaving structural elements, such as bronchidesmus and collagen tissue strands, intact. Two pairs of μEGG electrodes (see below) were inserted in tissue and fixed with 10/0 suture at locations indicated in Figs 1 and 2. For muscle stimulation experiments *ex vivo*, perfusion pathways were established in the zebra finch that allowed perfusion through the original syringeal vasculature. All arteriole capillaries were localized and closed using 10/0 suture immediately after exposure of the syrinx by a ventral incision to the sternum. The syrinx was then transferred to a Sylgard-covered petri dish on ice and connected to micro-perfusion lines, gravity fed with oxygenated Ringers. Perfusion pressure was kept at 2–4 kPa until patent perfusion was observed and immediately reduced to 1–2 kPa. The syrinx was typically mounted ventral side up in the experimental chamber to two bronchial and one tracheal connector (Supplementary Fig. 1).

### Experimental chamber design

To study syringeal sound production, we developed an experimental chamber that allowed for study of the syrinx *ex vivo* (that is, intact perfused organ) under controlled conditions. This experimental chamber was milled out of aluminium (temperature controlled) or PVC and covered by an airtight glass lid that allowed pressurization and a clear view (Supplementary Fig. 1). The glass was either coated with nano-particles (Percenta AG, Germany) or heated to 10 °C above chamber temperature by a 18-μm diameter Formvar coated Nichrome wire assembly (A-M Systems, Sequim, WA, USA), to prevent droplet forming fogging up the glass. In the floor centre of the chamber, a 1-mm raised edge with glass bottom covered in Sylgard allowed transillumination of the syrinx. We separately controlled pressure in the bronchial connectors (*p*_b_) and chamber (*p*_ICAS_) with dual valve differential pressure PID controllers (model PCD, 0–10 kPa, Alicat Scientific, AZ, USA), referenced to atmospheric pressure, and a response time of 1 ms. Bronchial mass flow was measured with micro-electro-mechanical system (MEMS) flow sensors of various ranges (PMF series, Posifa Microsystems, San Jose, USA) and a response time of 1 ms. The supply of pressurized air to the controllers was heated and humidified with deionised water in a pressure cooker. Excess vapour condensed in a downstream second pressure cooker. The pressure controllers were temperature controlled at 37 °C (HD4034 with PT100 sensor, Hotek Technologies, WA, USA) ensuring pre-heated, fully humidified air to the syrinx. The temperature of the aluminium experimental chamber was controlled with a recirculating water bath (Julebo F12-ED, Seelbach, Germany) and logged at 1 s intervals with a 1-mm diameter J-type miniature temperature probe (USBTC01, National Instruments, TX, USA) 1–2 mm away from the syrinx. Perfusion fluids attained the same temperature as the chamber due to physical contact with a platform in the chamber (labelled ‘Perfusate platform' in Supplementary Fig. 1). During the mounting process the temperature of the chamber was kept at 7 °C.

### Image acquisition and analysis

The syrinx was imaged through the chamber lid with a high-speed camera (MotionPro-X4, 12 bit CMOS sensor, Integrated Design Tools, Inc.; 4,000–25,000 frames s^–1^) mounted on a stereomicroscope (M165-FC, Leica Microsystems). For the zebra finch, we also used a more light-sensitive 16 bit high-speed camera (Fastcam SA1, Photron, San Diego, CA, USA; 20,000–35,000 frames s^−1^). We used a 1.2-mm (Schölly Fiberoptics, Denzlingen, Germany) and 300-μm (Advanced Inspection Technologies, Melbourne, FL, USA) diameter flexible fiberscope for endoscopic imaging. The low-light images obtained with the endoscopes were captured with a videokymographic (VKG) system (model 2156, Cymo B.V., Groningen, The Netherlands), which combines a high-speed linescan camera (7,200 line images s^−1^) with a full frame CMOS camera (25 frames s^−1^). The analogue video output from the VKG was captured together with the microphone and a synchronization signal using a capturing device (Intensity Extreme, Black Magic Design, Australia). Light for transillumination was provided by a 1,700 lumen LED (Luxeon S, Philips, The Netherlands) powered by a stable power source (PS23023, HQ Power, Belgium) or plasma light source (HPLS200, Thorlabs, Germany) through liquid light guides and reflected of a 45° angled silver coated prism (MRA series, Thorlabs) to absorb heat.

### Kymography

A kymogram displays pixel intensity over time along a set line in an image. The VKG system used here generates a kymogram at the fixed position of the linescan camera^63^. Digital kymograms (DKG) are kymograms along any arbitrary line in a digital image^64^ and were extracted from high-speed image recordings using Matlab (The Mathworks). Two kymograms in Supplementary Fig. 2 were previously published: human from ref. 40, and elephant from ref. 30.

### Micro-electroglottography

As an independent proxy for kinematics of the syringeal vibratory tissues, we used EGG. This non-invasive technique measures electrical impedance between electrodes and is commonly used to quantify vocal fold contact area in the larynx^65^. μEGG electrodes consisting of single stranded 25 μm diameter Formvar coated Nichrome wires (A-M Systems) were inserted as described above and connected to a modified two-channel electroglottograph (model EG2, Glottal Enterprises Inc. NY, USA).

### Data acquisition and synchronisation

Sound was recorded with a ½ inch pressure microphone-pre-amplifier assembly (model 46AD with preamplifier type 26AH, G.R.A.S., Denmark), amplified and high-pass filtered (10 Hz, 3-pole Butterworth filter, model 12AQ, G.R.A.S.). The microphone sensitivity was measured before each experiment (sound calibrator model 42AB, G.R.A.S.). The microphone was placed at 2–3 cm from the tracheal connector outlet in the acoustic near field, and on a 45° angle to avoid the air jet from the tracheal outlet. The sound signal was time shifted for the travelling distance from vibratory membranes to microphone. Microphone, μEGG, pressure and flow signals were low-pass filtered at 10 kHz (custom-built filter). These signals together with synchronization signals from camera systems and muscle stimulators were digitized at 50 kHz (USB 6259, 16 bit, National Instruments, Austin, TX, USA). These signals were synchronized with all imaging systems with an accuracy of <21 μs before each experiment. All control and analysis software was written in Labview (National Instruments) or Matlab.

### Tissue-wave imaging protocol and analysis

Transillumination successfully visualized the inside outlines of vibratory tissues in the domestic pigeon, Barbary dove, elegant-crested tinamou and zebra finch. High optical density of the ostrich and cockatiel syrinx did not allow transillumination. In the tinamou, we dissected apart *M. syringealis* to allow LVMs imaging using transillumination. In zebra finch transillumination was successful in adult females and juvenile males, but attempts in adult males were unsuccessful due to the high optical density of *M. syringealis ventralis* (VS) muscles. In the zebra finch sound production was induced in the right hemisyrinx. Substantially different requirements in lighting conditions did not allow for simultaneous transillumination and tracheal endoscopy in zebra finches.

To image syringeal oscillatory behaviour we subjected the syrinx to a single bronchial pressure ramps at constant air sac pressure, while filming from 5,000 to 35,000 frames s^−1^. Our system could acquire and save a maximum of 2–10 s of data depending on frame rates, which comprised (tens of) thousands of frames. Due to the image complexity of the data, analysis could not be automatized, and required frame-by-frame manual analysis. On selected sequences of high-speed images, the left and right LVM or labia were traced manually in Amira (Visage Imaging GmbH, Berlin, Germany) and processed in Matlab. We calculated displacement (for example, Fig. 1e top panel) as current position minus the most lateral position during oscillation (indicated by the green line ‘min' on the top left in Fig. 1e). With both left and right LVM shapes quantified we could then compute the syringeal opening as a function of position and time, a graph also known as the glottovibrogram^30^ as can be seen in Figs 1e, 2d and 3d.

To quantify syringeal dynamics over a range of F0 values, full glottovibrogram reconstructions over several cycles were not suitable due to the labour intensive manual tracing required. Therefore we used an alternative approach to determine the presence of the VC2 component and speed of the tissue wave across a range of F0 values. For pigeon, tinamou and zebra finch we used transilluminated frontal views of the syrinx and calculated one DKG at the caudal edge and one DKG at the cranial edge of the LVM or labia (top two panels in Fig. 1d) at distance Δ*y* (indicated in middle panel Fig. 1c). DKG's contain many periodic traces as many structures move with each oscillation. By tracing selected LVM and labial inner edges in video stills over five oscillation cycles, we could identify the wave representing the motion of the LVM or labial inner edge in the DKG's and as such measure the time difference between maximal lateral position of LVM or labium on the two DKG's (Δ*t*_DKG_ in Fig. 1d). The wave speed equalled *v*_VC2_=Δ*y*/Δ*t*_DKG_ and was averaged over five consecutive cycles. Because in the cockatiel syrinx transillumination was not successful, we used cross-correlation between two μEGG electrode pairs located at distance Δ*y* to calculate time delay (Δ*t*_μEGG_) and wave speed *v*_VC2_. The ostrich was not included in this analysis because both transillumination was not successful and μEGG did not resolve vibrations due to lack of syringeal closure. We did not estimate wave speed from the tracheal endoscopic view because the VC2 velocity component was almost normal to the imaging plane, leading to large inaccuracies.

In these five consecutive oscillations, we quantified the timing of syringeal opening and closing from the transilluminated frontal views as the first frame in which the entire syringeal passage was open or closed, respectively. The minimal precision for these measurements equals the duration of one frame as indicated by the black vertical line at the base of each column in Fig. 5. In addition, we quantified the timing of acoustic excitations in the sound pressure signal. The timing of the sound signals was corrected for the time needed for the sound to propagate from the oscillators through trachea and tubing to the microphone, assuming a sound speed of 340 m s^−1^. With these parameters we quantified the timing of acoustic excitation with respect to syringeal opening and closing events within single oscillatory cycles.

### Pressure control space protocol

To explore syringeal oscillatory behaviour in the pressure control space we subjected the syrinx to a set of bronchial pressure ramps at randomized and variable air sac pressure, and one additional ramp where *p*_b_ equalled *p*_ICAS_. Ramp speed was 1 kPa s^−1^. Depending on species, we used pressures <2.0–4.0 kPa and |*p*_b_−*p*_ICAS_|<2.0 kPa to avoid regimes of high flow (that is, combinations of high *p*_b_ and low *p*_ICAS_) and/or mechanical failure of syringeal oscillatory structures. These sets were performed without transillumination imaging. SPL and WE were calculated on 100 ms segments by applying a sliding window with 50 ms steps. If the SPL of these segments were above the 60 dB re. 20 μPa threshold, segments were zero-padded to the next power of two, and a 4,096 point power spectral density estimate was computed (periodogram method in Matlab). Additional filtering was used to extract the fundamental frequency (F0) reliably from the spectral density estimate. WE was calculated as the geometric mean of the power spectrum over the arithmetic mean of the power spectrum.

We extracted iso-F0 contours in *p*_b_, *p*_ICAS_ control space. SPL and WE were evaluated along the iso-F0 contours at a resolution of 10 Pa (Supplementary Fig. 3). To evaluate whether points on these iso-F0 contour lines were redundant for all three quantified acoustic parameters (F0, SPL and WE) we computed acoustic parameters at a fixed distance of 100 Pa both before and after each point along the iso-F0 contour line locations in *p*_b_, *p*_ICAS_ control space (Supplementary Fig. 3). For the 100 Pa start and end sections of the contour, only forward and backward values could be computed. A point along the iso-F0 contour was considered redundant for all the three parameters if the WE and SPL variation within the 100 Pa interval was below 0.2 units of WE and 1 dB SPL (ref. 66). This resulted in a logical (1/0) redundancy array for each iso-F0 contour. The iso-F0 contour is plotted white when redundant and black when not in the *p*_b_, *p*_ICAS_ control spaces in Figs 6a–d and 7c.

We computed iso-F0 contours at fixed intervals (*Columba*, 10 Hz; *Streptopelia*, 50 Hz *Eudromia*, 50 Hz; *Nymphicus*, 100 Hz; *Lonchura*, 100 Hz; *Taenopygia*, 50 Hz) within the F0 range observed in the preparation. The percentage redundancy of a pressure control space was expressed as the ratio of redundant points to the total amount of points on all iso-F0 contours computed in the *p*_b_, *p*_ICAS_ control space.

### Muscle stimulation protocol

Muscles were stimulated with 50 μm diameter Teflon-coated twisted silver wire electrodes (A-M Systems) that were fixed with 10/0 suture. We used a stimulus isolator (model A395, WPI, Sarasota, FL, USA) to apply variable currents (0–10 mA) when limited compliance voltage was sufficient to contract muscles (tinamou). For zebra finches and Bengalese finches, we used two stimulators (model 14E11, DISA, Herlev, Denmark and model 2100, A-M Systems, respectively). To ensure muscle specificity, we placed carbon microspheres (20–40 μm diameter) at regular intervals on the muscles and surrounding tissues and filmed contractions at 1 kHz. We stimulated the single syringeal muscle present in tinamous, *M. syringealis*. In songbirds, activity of the *M. syringealis ventralis* correlates to F0 (ref. 67). Therefore we focussed on this muscle to study F0 control in zebra finches (*N*=5 adult males). F0 of the sound was determined 30 ms prior (control) and 30 ms after stimulation. We computed 512-point fast Fourier transforms and used parabolic interpolation around the peak power to determine F0.

### Statistics

Linear regressions were calculated to test the significance of wave speed as a function of F0 in Matlab. All values are presented as mean±1 s.d.

### Morphome reconstruction

We constructed three-dimensional annotated syrinx morphologies, that is, morphomes, based on IKI contrasted CT scans^56^. All specimens were scanned at Odense University Hospital with a μCT scanner (VivaCT 40, Scanco Medical AG, Switzerland) with 10 μm resolution, except the ostrich syrinx which was scanned with 80 μm resolution on a XtremeCT (Scanco Medical AG). These scans were annotated manually in Amira for bone, soft tissue and muscle using protocols cf. ref. 56.

### Natural vocalizations

Sound recordings of natural vocalizations were obtained in previous studies for domestic Barbary dove^7^, elegant-crested tinamou^68^ and zebra finch^69^. A domestic pigeon call recording was obtained from www.xeno-canto.com (catalogue nr: XC92264). We recorded the mating calls of two male ostriches at an open-range ostrich-breeding farm (Langeskov, Denmark) from April to May 2013 using a shotgun microphone (model KMR82i, Neumann, Berlin, Germany) and a 24-bit digital recorder (Olympus LS-100). Cockatiel recordings were made with the same shotgun microphone and solid-state recorder (model PMD-670, Marantz, Mahwah, NJ, USA).

## Additional information

**How to cite this article:** Elemans, C. P. H. *et al.* Universal mechanisms of sound production and control in birds and mammals. *Nat. Commun.* 6:8978 doi: 10.1038/ncomms9978 (2015).

## Supplementary Material

Supplementary Information Supplementary Figures 1-3

Supplementary Movie 1 High-speed video of syringeal kinematics during sound production in the domestic pigeon. The first part of the video is imaged under incident lighting, followed by trans-illumination, which clearly reveals the inner contours of lateral vibratory mass (LVM) motion. The visible wires are 18 μm diameter nichrome μEEG wires. Recorded at 4000 frames s^-1^ and slowed 133 times. The extracted LVM inner contours were superimposed upon the individual images (right, green; left, red)

Supplementary Movie 2 High-speed video of syringeal kinematics during sound production in the elegant crested tinamou. Trans-illumination clearly reveals the inner contours of LVM motion. The visible wires are 18 μm diameter nichrome μEEG wires. Recorded at 4000 frames s^-1^ and slowed 133 times.

Supplementary Movie 3 High-speed video of syringeal kinematics during sound production in the Barbary dove. Trans-illumination clearly reveals the inner contours of LVM motion. The visible wires are 18 μm diameter nichrome μEEG wires. Recorded at 5000 frames s^-1^ and slowed 167 times.

Supplementary Movie 4 High-speed video of syringeal kinematics during sound production in the ostrich. Tracheal endoscopic view shows presence of both caudocranial as well as ventrodorsal waves. Recorded at 1000 frames s^-1^ and slowed 67 times.

Supplementary Movie 5 High-speed video of syringeal kinematics during sound production in the cockatiel. Recorded at 5000 frames/s and slowed 167 times. The morphology of this syrinx does not allow transillumination, but the tracheal endoscopic view clearly shows LVM kinematics during opening and closing of the. Recorded at 5000 frames s^-1^ and slowed 670 times.

Supplementary Movie 6 High-speed video of syringeal kinematics during sound production in the zebra finch. Trans-illumination reveals the inner contours of the left medial labium (ML) motion. The visible wires are 18 μm diameter nichrome μEEG wires. Recorded at 2000 frames s^-1^ and slowed 335 times. The extracted ML contours (green) were superimposed upon the individual images.
