# Supplementary material for: Universal mechanisms of sound production and control in birds and mammals
Source: Nat Commun. 2015 Nov 27;6:8978. doi: 10.1038/ncomms9978 (PMC4674827; doi:10.1038/ncomms9978)
Supplement: Supplementary Information — Supplementary Figures 1-3 [file ncomms9978-s1.pdf]

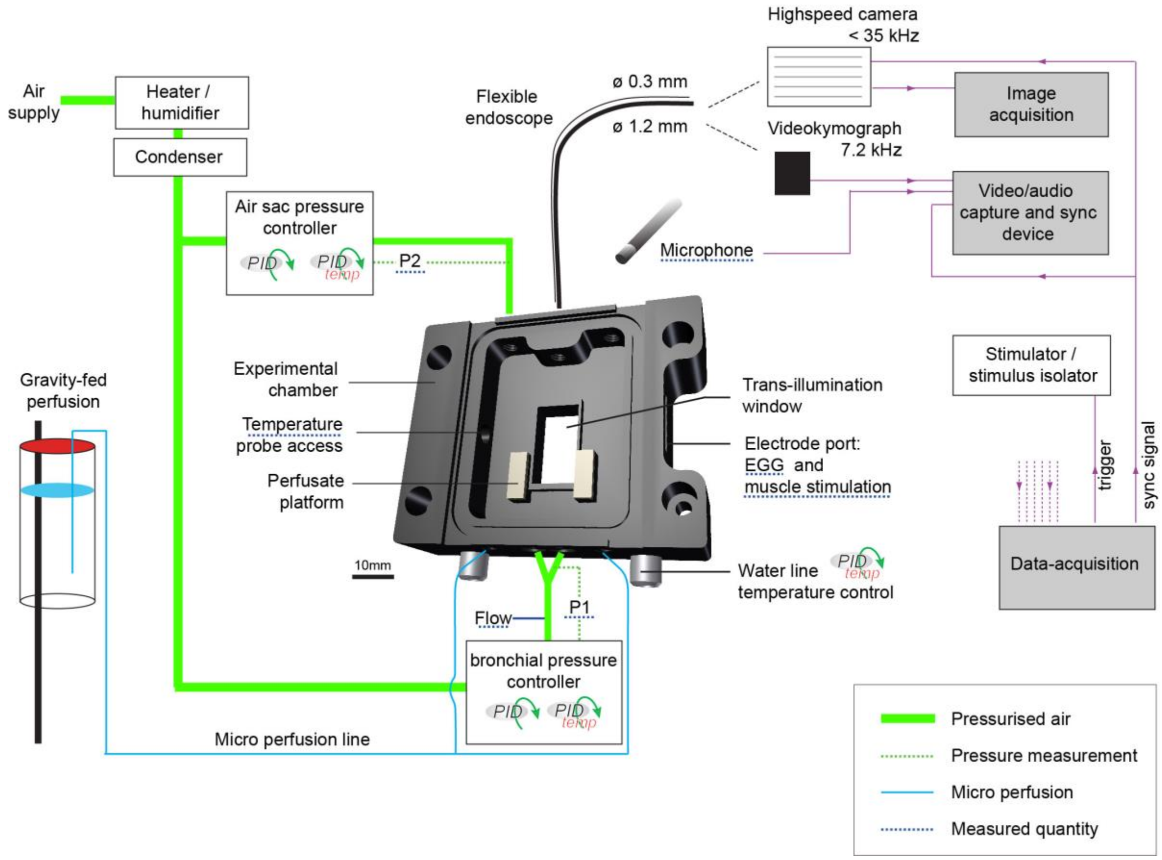

Supplementary Figure 1Schematic representation of experimental chamber, setup and data-acquisition. P1, bronchial pressure; P2, air sac pressure. PID controller, proportional-integral-derivative controller.

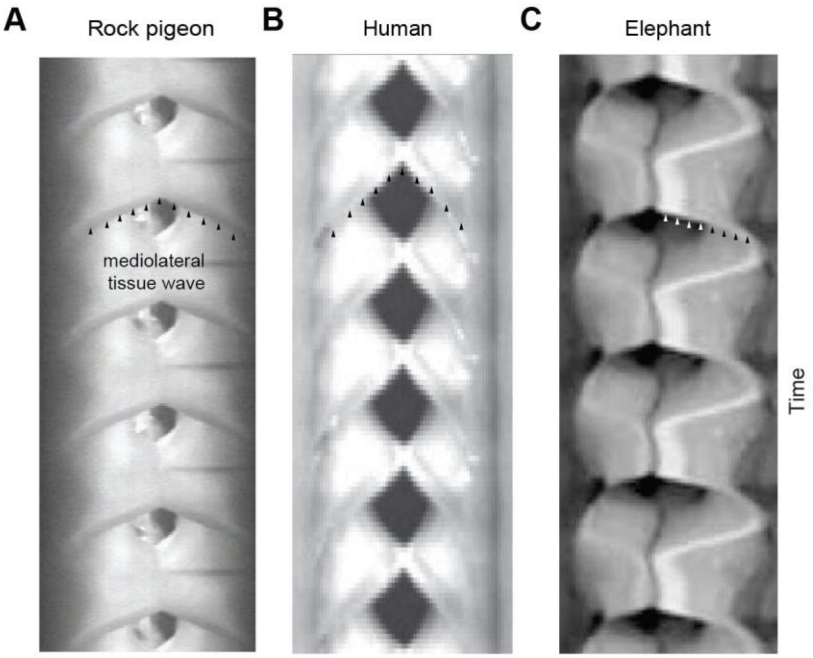

Supplementary Figure 2Mediolateral tissue waves are present in birds and mammals. Endoscopic videokymograms of domestic pigeon syrinx, human larynx[40] and elephant larynx[30] show presence and similarity of laterally travelling mucosal wave upon syringeal and glottal opening.

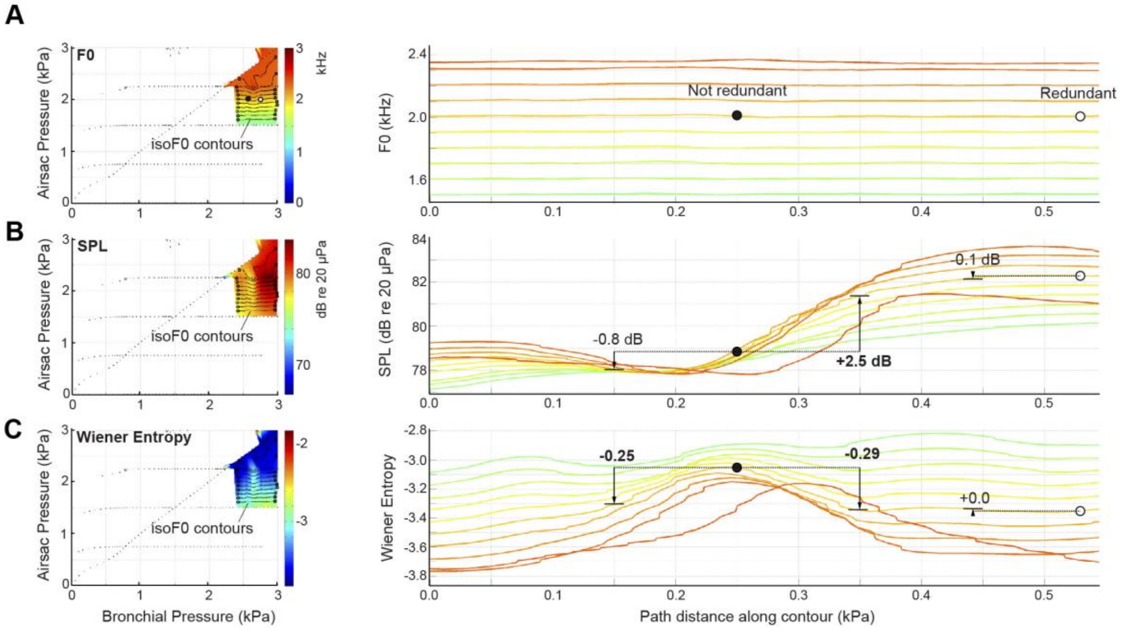

Supplementary Figure 3

Example of redundancy computation. Interpolated contour plots (left) of the extracted **(a)** F0, **(b)** SPL, and **(c)** Wiener entropy (WE) of sounds measured in the pressure control space. This example uses Bengalese finch left hemi-syrinx data shown in Fig. 6D. Iso-F0 contour lines are shown on each contour plot (black lines with circle at start and square at end). The values of F0, SPL and WE are evaluated along the iso-F0 contours (right panels), color-coded as in the top-left F0 contour plot. Thus the green to yellow to red lines correspond to the iso-F0 contours of 1500 to 2350 Hz in steps of 100 Hz in all three right panels. A point along any iso-F0 contour is considered not redundant (black filled circle) if SPL and WE cross the set threshold (1 dB and 0.2 units, respectively) when moving 100 Pa in both directions along the iso-F0 contour (see methods). For example, the SPL values at 100 Pa distance along the iso-F0 contour after the filled black circle changes more than 1dB (+2.5. dB) (middle, right panel) and WE changes more than 0.2 units (-0.25 at 100 Pa before and -0.29 at 100 Pa after). This point is thus considered not-

redundant. A point along any of the iso-F0 contours is considered redundant (white circle) if SPL and WE remain below threshold at 100 Pa distance. The redundant example shown is within 100 Pa to the right border of the iso-F0 contour and thus only the values a 100 Pa left of the point are taken into consideration (see methods).
